# The (In)dependence of Alternative Splicing and Gene Duplication

**DOI:** 10.1371/journal.pcbi.0030033

**Published:** 2007-03-02

**Authors:** David Talavera, Christine Vogel, Modesto Orozco, Sarah A Teichmann, Xavier de la Cruz

**Affiliations:** 1 Molecular Modeling and Bioinformatics Unit, Parc Científic de Barcelona, Barcelona, Spain; 2 Protein Structure and Modelling Node, Instituto Nacional de Bioinfomática, Genoma España, Parc Científic de Barcelona, Barcelona, Spain; 3 Medical Research Council Laboratory of Molecular Biology, Cambridge, United Kingdom; 4 Institute for Cellular and Molecular Biology, University of Texas Austin, Austin, Texas, United States of America; 5 Departament de Bioquímica i Biologia Molecular, Universitat de Barcelona, Barcelona, Spain; 6 Computational Biology Program, Barcelona Supercomputing Center, Barcelona, Spain; 7 Institut per a la Recerca i Estudis Avançats (IRCA), Barcelona, Spain; University of California San Diego, United States of America

## Abstract

Alternative splicing (AS) and gene duplication (GD) both are processes that diversify the protein repertoire. Recent examples have shown that sequence changes introduced by AS may be comparable to those introduced by GD. In addition, the two processes are inversely correlated at the genomic scale: large gene families are depleted in splice variants and vice versa. All together, these data strongly suggest that both phenomena result in interchangeability between their effects. Here, we tested the extent to which this applies with respect to various protein characteristics. The amounts of AS and GD per gene are anticorrelated even when accounting for different gene functions or degrees of sequence divergence. In contrast, the two processes appear to be independent in their influence on variation in mRNA expression. Further, we conducted a detailed comparison of the effect of sequence changes in both alternative splice variants and gene duplicates on protein structure, in particular the size, location, and types of sequence substitutions and insertions/deletions. We find that, in general, alternative splicing affects protein sequence and structure in a more drastic way than gene duplication and subsequent divergence. Our results reveal an interesting paradox between the anticorrelation of AS and GD at the genomic level, and their impact at the protein level, which shows little or no equivalence in terms of effects on protein sequence, structure, and function. We discuss possible explanations that relate to the order of appearance of AS and GD in a gene family, and to the selection pressure imposed by the environment.

## Author Summary

Alternative splicing (AS) and gene duplication (GD) followed by sequence divergence constitute two fundamental biological processes contributing to proteome variability. The former reflects the ability of many genes to express different products, while the latter results in several copies of the same gene that are similar but not identical. In spite of these obvious differences, recent computational studies as well as anecdotal experimental evidence suggested that AS and GD produce functionally interchangeable protein variants. We provide a detailed study of the differences between alternative splicing and gene duplication and discuss potential interchangeability with respect to variation in expression, protein structure, and function. In general, the contribution of these two processes to the proteome variability is substantially different, and we advance some explanations that may explain this apparent contradiction and contribute to our understanding of the evolution of complex, eukaryotic proteomes.

## Introduction

**Figure 1:**
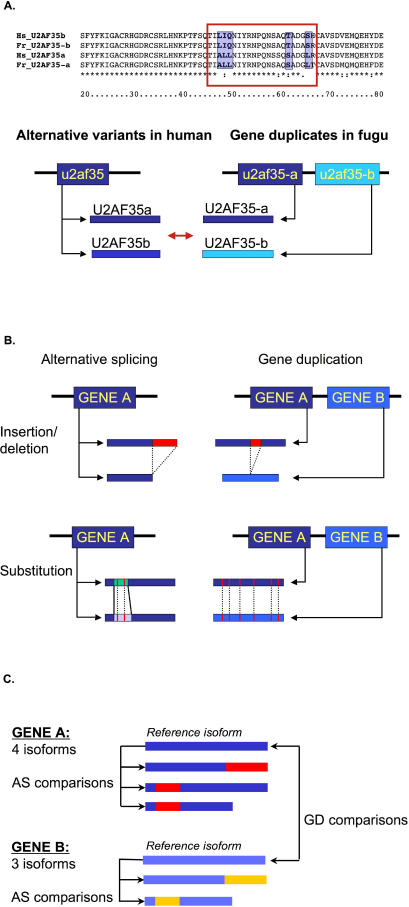
Equivalence between Alternative Splicing and Gene Duplication (A) The alignment shows an example of molecular equivalence between the effects of AS and GD. The human U2AF35 gene has two known splice variants, Hs_U2AF35a and Hs_U2AF35b, that differ along the region marked with a red box. The fugu orthologue Fr_U2AF35-a does not have known splice variants, but instead has a paralogue, Fr_U2AF35-b [9]. All sequences have kindly been provided by T. R. Pacheco and M. Carmo-Fonseca. For some residues (bold, highlighted in light blue), the substitutions amongst the human splice variants are equivalent to those in the fugu GD. The cartoon illustrates the relationship between the human and fugu sequences. The names of genes and their protein products are denoted in small and capital letters, respectively. At the molecular level, AS and GD show equivalent changes to sequence, and therefore are likely to have interchangeable effects on structure and function of the proteins. In this work we study whether such molecular interchangeability holds in general. (B) We compared the characteristics of two types of sequence changes, indels and substitutions, between AS (both shown in dark blue) and GD (shown in dark and light blue). On top, we illustrate an indel event (the deleted stretch is highlighted in red, and two dotted lines denote its location); at the bottom, we illustrate substitution events (red lines represent residue matches between sequences, linked by dotted lines; the continuous lines between alternative splice isoforms represent the boundaries of the interchanged stretches). (C) We used this protocol in all sequence comparisons between AS and GD. Changes between alternative splice isoforms are obtained after comparing the SwissProt [44] reference isoform with the remaining isoforms. Changes between duplicates are obtained by comparing the SwissProt [44] reference isoforms of the genes that are part of one GD family.

Alternative splicing (AS) and gene duplication (GD) are two main contributors to the diversity of the protein repertoire with enormous impact on protein sequence, structure, and function [1–5]. Interestingly, several recent studies point to a direct equivalence between AS and GD. There are some cases where alternative splice variants in one organism are similar to gene duplicates in another organism [6–9]. For example, the eukaryotic splicing factor U2AF35 has at least two functional splice variants in human, U2AF35a and U2AF35b, which differ by seven amino acids in the RNA recognition motif (Figure 1A). The fugu orthologue U2AF35-a has no splice variant; instead there is a duplicate gene U2AF35-b with changes identical to those found in the human splice variant U2AF35b [9].

Further, the changes introduced to a sequence are constrained by the need to preserve a stable and functional three-dimensional (3-D) fold [10]. Indeed, structural studies have shown that insertions and deletions between gene duplicates tend to happen at sequence locations where they are less damaging [11], such as loops at solvent-accessible locations. These restrictions will apply irrespective of the source of the changes and thus may introduce a certain degree of similarity between the sequence changes associated with GD and AS. Finally, recent studies have shown that AS and GD are inversely correlated on a genome-wide scale [12,13], i.e., small gene families tend to have more genes with alternative splice variants than do large families. These findings together—i.e., anecdotal examples, structural constraints, and anticorrelation at the genomic level—suggest that AS and GD are interchangeable sources of functional diversification [12]. Genes with AS would not need to produce additional variants in the form of duplicates, and vice versa*.*

Here, we first tested the anticorrelation between AS and GD with respect to sequence divergence, function, and gene expression. Second, we studied the interchangeability hypothesis at the protein structure level and asked to what extent AS and GD introduce changes to the sequence that are equivalent in their nature and effect on structure and function. To this end, we conducted a large-scale comparison of the effects of AS and GD on human and mouse proteins (Figure 1B and 1C; the results for the analysis of the mouse data can be found in Figure S4). For the vast majority of cases, the two processes result in different protein modifications with different functional implications. This finding, while consistent with the different molecular mechanisms underlying both phenomena, contradicts the anticorrelation observed at the genomic level. We discuss some possible explanations for this paradox.

## Results/Discussion

### Genomic Analysis

**Figure 2:**
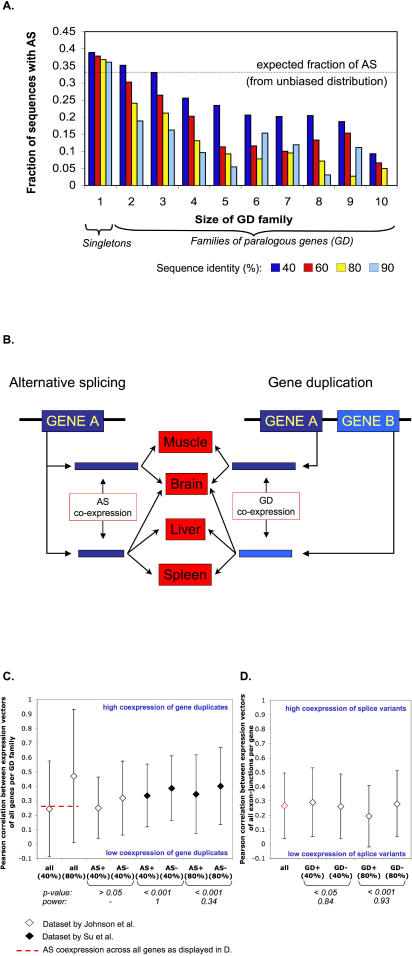
The Relationship between AS and GD at the Genomic Level (A) The diagram shows the uneven distribution of AS amongst GD families of different sizes for the human genome. Information on AS has been taken from the AltSplice database [43]. GD families were obtained by clustering all sequences of more than 40%, 60%, 80%, or 90% seq.id., respectively, using CD-HIT [47]. The dashed line marks the expected fraction of genes with AS, given an unbiased distribution of all known genes with splice variants across the whole genome. In accordance with previous results [12,13], for large GD families we observe fewer genes with AS than expected at random. (B) The cartoons illustrate that alternative splice isoforms and gene duplicates may be expressed in the same number and/or types of tissues. Here, we compared the extent of coexpression amongst alternative splice variants (AS coexpression) and gene duplicates (GD coexpression). (C) Coexpression levels amongst gene duplicates (GD coexpression) are estimated as the average pairwise PC between expression patterns of all genes within a GD family. GD coexpression amongst duplicates of >40% seq.id. (white diamonds) is more similar to the overall AS coexpression (red line indicating the value displayed in Figure 2D) than GD coexpression amongst duplicates of >80% seq.id. In other words, coexpression of alternative splice variants is similar to coexpression amongst gene duplicates of >40% seq.id. As this dataset [17] is too small for GD80 families to be split into further subsets, we examined GD coexpression in an additional dataset [53] (black diamonds). For both 40% and 80% seq.id., expression variation amongst gene duplicates with alternative splice variants (AS+) is slightly higher than variation amongst gene duplicates without alternative splice variants (AS−). *p*-Values are based on *t*-test calculations. Data on alternative splice variants was taken from the AltSplice database [43]. Further details and results are provided in Table S4 and Figure S10A and S10B. (D) Coexpression levels amongst alternative splice variants (AS coexpression) are estimated as average pairwise PC between the expression patterns of all exon junctions of a gene. High PC indicates little variation (high coexpression), and vice versa. The figure shows average AS coexpression across all genes in the dataset [17], and across subsets of the genes: GD families (GD+) and singletons (GD−) as defined by >40% and >80% seq.id., respectively. The overall AS coexpression is marked as a red diamond and indicated as a red line in Figure 2C. Further details are provided in the Table S4 and Figure S10A and S10B. *p*-Values are based on *t*-test calculations. Gene duplicates of high seq.id. (>80%) have slightly lower AS coexpression than singletons (*p*-value < 0.001).

In accordance with recent findings [12,13], AS and GD are anticorrelated at the genomic level (Figure 2A). There are fewer alternatively spliced genes in large families of gene duplicates than expected in an unbiased distribution. This depletion is strongest in gene families of high sequence identity (seq.id) (>80% seq.id., GD80, Figure 2A), and weaker but still present for more diverged families (>40% seq.id, GD40). The same trend holds true when examining orthologues: the bias is stronger amongst human–mouse or human–fly orthologues (GD80, χ^2^-value = 150 and χ^2^-value = 105, respectively; *p*-value ≪ 0.001 in both cases) than amongst human–yeast orthologues (GD80, χ^2^-value = 84; *p*-value ≪ 0.001).

#### Gene function.

The anticorrelation between AS and GD could arise from the preferential duplication or introduction of AS in genes of particular function. In general, genes with AS have similar distributions across functional categories as genes with GD (see Table S3, Figure S2), with two exceptions. Ribosomal and receptor proteins (e.g., G protein–coupled receptors) belong to the largest protein families in human [14], and thus their enrichment in families of GD is unsurprising (E-value < 10^−10^). At the same time, these functions are depleted amongst genes with alternative splice variants [15], which is also reflected in our data (E-value < 10^−10^).

When removing from our dataset the 855 and 293 proteins predicted to be G protein–coupled receptor-like or ribosomal proteins [14], respectively, the bias against genes with both AS and GD (AS+/GD+) is still strongly significant (*p*-value ≪ 0.001). See Table S3 for details. Thus, the anticorrelation between AS and GD is not due to biases amongst genes of different functions.

#### Variation in expression patterns.

We further characterized the relationship between AS and GD by comparing their patterns of expression among different tissues, which reflect corresponding regulatory processes. A previous study reported that AS and general transcription regulation act independently on different groups of genes with tissue-specific expression [16]. Here we do not compare tissue specificity, but the overall extent of variation in expression introduced by AS and GD. More precisely, we studied whether the extent of coexpression (i.e., the lack of expression variation) between alternative splice isoforms is comparable to that found between duplicates (Figure 2B). If AS and GD are functionally interchangeable, then we would expect a similar amount of coexpression amongst their products.

The level of coexpression was measured using the Pearson correlation coefficient (PC) between the expression patterns of two isoforms among a set of tissues, in the case of AS, or of two duplicates, in the case of GD. When more than two isoforms (or duplicates) were available, we averaged the PCs resulting from all the possible comparisons between them (see Material and Methods and Figure S10). PC values near 1 or 0 correspond to high or low coexpression, respectively.

We directly compared the coexpression in alternative splice variants (AS coexpression) and in gene duplicates (GD coexpression) using data from the same microarray experiment [17] (white diamonds in Figure 2C–2D). We observe that the overall AS coexpression (PC = 0.27 ± 0.23) is comparable to the overall GD coexpression of GD families with >40% sequence similarity (PC = 0.24 ± 0.33) (Figure 2C–2D, red line and diamond). This suggests that AS and GD40 may have comparable levels of coexpression—that is, they both span similar ranges of tissue expression, though the underlying molecular regulation generating coexpression of AS isoforms and GD40 transcripts is different. However, due to the relatively small size of the dataset and the use of exon junctions as a proxy for alternative splice variants, this result should be treated with care.

We also explored whether we can observe, at the expression level, an anticorrelation in analogy to that found at the sequence level [12,13] (Figure 2A). A previous study has shown that duplicates diverge quickly in their expression patterns [18], so we would expect a smaller amount of GD coexpression for genes without AS (AS–/GD+) than for those with AS (AS+/GD+). However, we find the opposite (Figure 2C), although the trend is very weak and in most cases the statistical power is low. This reverse trend suggests that in AS+/GD+ families, expression variation amongst alternative splice isoforms may contribute independently of the GD expression variation to the overall expression variation amongst all AS and GD variants of the gene; we observe some additivity rather than complementarity. Figure 2D confirms this finding for AS coexpression: there is no obvious anticorrelation between AS and GD in terms of their effect on expression variation amongst their variants.

We note that the coexpression analysis is at present still limited by the amount of data available. Future availability of large-scale datasets suited for expression comparison of AS and GD will help refine our results.

### Protein Sequence and Structure Analysis

To test whether AS and GD are interchangeable at the structural and thus functional level, we compare sequence changes between duplicate proteins to those between alternative splice isoforms (Figure 1B–1C). There are two basic types of sequence changes in both AS and GD: substitutions, and insertions or deletions (indels) (Figure 1B). Both can be described in terms of their length, the nature of the affected amino acid residues, and their location in the structure or relative to each other in the sequence. We use these properties as changes in their values can be directly related to changes in protein structure and function [19]. We analyzed the properties strictly from a protein structure and function point of view; we do not examine the evolutionary processes (such as selection) that led to them.

We focus on gene families defined using two seq.id. thresholds: 80% and 40%. The former was chosen because the anticorrelation at the genomic level is stronger (Figure 2A), and global seq.id. between alternative splice variants is >80% (see below). However, GD data at the 80% seq.id. level may display only a few sequence changes, resulting in larger than expected differences between AS and GD. To avoid this bias we have also considered more diverged families, defined by a 40% seq.id. threshold. Figure S5 contains additional analyses on GD, using families defined by common protein domains. In our analyses described below, we focus on gene families that have both alternative splice variants (AS+) and gene duplicates (GD+), i.e., AS+/GD+, except in the case of local sequence identities, for which we also extend our study to families AS–/GD+ and AS+/GD–.

### Substitutions

First we examine substitutions, i.e., the extent and nature of amino acid changes and the length of the substituted region. In general, substitutions amongst GD range from a small number of amino acid replacements in recent homologues to a large number of replacements in proteins as divergent as haemoglobin, for instance [10]. In AS, substitutions in one isoform as compared with another one arise by the use of mutually exclusive exons [20], although they can also be due to intron retentions accompanied by stop codons [21].

#### Global versus local sequence identity.

Global seq.id. is the seq.id. along the whole alignment of two sequences, and it can be used to assess the overall degree of function conservation between proteins [22]. However, same global seq.id. values may correspond to very different distributions of amino acid replacements along the sequence. For this reason, we also examined local seq.id., i.e., the seq.id. between only parts of two sequences. For alternative splice variants, the local seq.id. corresponds to substituted regions, i.e., mostly mutually exclusive exons. For GD, we estimated the corresponding sequence stretches by different methods as described below.

**Figure 3:**
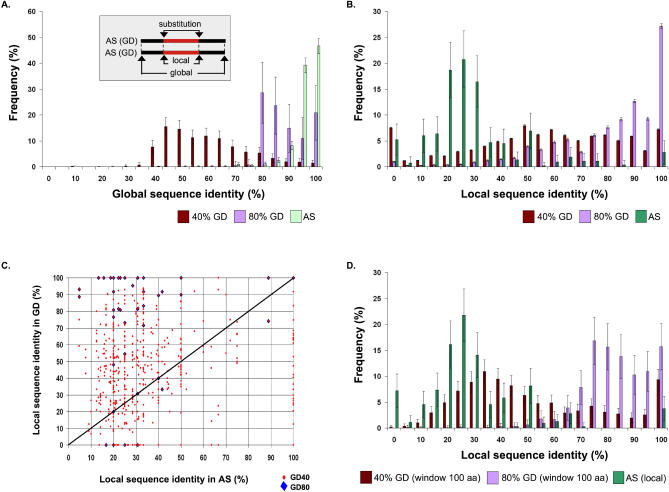
Global and Local Sequence Identity in AS and GD Substitutions AS data were obtained by querying SwissProt [44] database version 40, with the keywords VARSPLIC and HUMAN. GD data were obtained by clustering the SwissProt [44] data using CD-HIT [47] to 40% or 80% seq.id. (GD40 and GD80, respectively). We focus on AS+/GD+ cases, i.e., those sequences with both AS and GD, in Figure 3A–3C, and discuss the AS−/GD+ versus AS+/GD− case in Figure 3D. (A) Global seq.id. The seq.id. in GD families depends on the cutoff used for clustering, e.g., GD40 (dark red) or GD80 (light violet), respectively. The global seq.id. between alternative splice isoforms (light green) is very high ( >90% seq.id.), reflecting the underlying nature of AS changes. (B) Local seq.id. in alternative splice isoforms (dark green) is measured between substituted stretches, usually arising from mutually exclusive exons. The local seq.id. between gene duplicates is obtained using a moving window (GD80: light violet, GD40: dark red) and reporting the seq.id. observed in all possible window positions. (C) Local seq.id. in AS and GD at equivalent positions. The graph compares local seq.id. found in alternative splice variants of a gene with the local seq.id. of a duplicate of the same gene. The AS local seq.id. was computed between substituted sequence stretches. For GD, we mapped the sequence positions of the AS event to the aligned GD, and computed the seq.id. between the GD, considering only the aligned positions within that region. The comparison is shown for AS and GD40 (red) and GD80 (blue), respectively. The diagonal separates the plot into two halves: the upper half corresponds to the region for which GD seq.id. is higher than that for AS; the lower half corresponds to the opposite. For both types of gene families (GD40 and GD80), most substitutions show higher seq.id. amongst gene duplicates than amongst alternative splice variants, and this bias is significant (GD80: 111 of 142, χ^2^ test *p*-value < 1.9 × 10^−11^; and 492 of 786, χ^2^ test *p*-value < 6.5 × 10^−15^, respectively). This result confirms the overall distributions examined in Figure 3B: changes in AS are stronger and more localized than those in GD. (D) Local seq.id. in AS−/GD+ and AS+/GD− substitutions. To compute local seq.id. in AS−/GD+ families, we first align two GD, then slide a 100-aa window over the sequence of one protein, and compute the seq.id. at all sequence positions of the window. The results of all the possible comparisons are plotted for GD40 (dark red) and GD80 (light violet) families. For genes with AS but no duplicates (AS+/GD−) (dark green), local seq.id. was computed between the two substituted stretches resulting from AS events. As for AS+/GD+ families (Figure 3B), we find that, in general, local seq.id.s are substantially lower for AS events (AS+/GD−) than for GD (AS−/GD+ families). The overlap between the AS and GD40 families is higher than that between AS and GD80 families, which may partly be due to differences in the structure constraints applying to the proteins in each set.

In Figure 3 we show the distributions of global and local seq.id. for AS and GD. In the case of AS we see that global seq.id. (Figure 3A) between splice isoforms is very high (>90%), but local seq.id. (Figure 3B) between the substituted segments in splice isoforms is low (<30%). This is in accordance with the underlying molecular mechanisms of AS [20,21], which point to very localized sequence changes. The global seq.id. for GD families are broadly distributed above the respective seq.id. cutoff (Figure 3A).

In contrast, while the local seq.id. in alternative splice variants is usually low, it is clearly higher for GD, in particular for GD80 families (Figure 3B). This result implies that at comparable global seq.id. (i.e., >80%; see Figure 3A), alternative splice variants have more local changes than gene duplicates. The differences are smaller, although still clear, if we consider local seq.id. GD40 families.

Global and local seq.id. provide a first view on interchangeability between AS and GD. However, to understand the effects of substitutions, it is also important to know the location of the changes [23,24]. To retrieve such information, we directly compared seq.id. between substituted regions in AS with the equivalent regions of their gene duplicates (AS+/GD+, Figure 3C). In the majority of cases, local seq.id. for GD is higher than the local seq.id. of AS (GD80: 78%, χ^2^-test *p*-value < 1.9 × 10^−11^; GD40: 64%, χ^2^-test *p*-value < 6.5 × 10^−15^).

In general, we find that the distribution of amino acid replacements along the protein sequence is different between AS and GD substitutions. In gene duplicates, changes are spread all over the sequence, while in alternative splice variants the changes are concentrated at very precise locations, in accordance with the underlying molecular mechanisms [20,21]. In general, these data point to a noninterchangeability between AS and GD changes. However, when the sequence divergence between duplicates is large, as is the case for GD40 families, there may be some cases for which AS and GD changes have comparable impact on protein function.

The comparisons described above have been derived from gene families with alternative splice variants and gene duplicates (AS+/GD+, Figure 3A–3C). Next, we extend our analysis to genes for which only either AS or GD, but not both, are known to exist (AS+/GD– and AS–/GD+, Figure 3D). Because AS information is not available for the AS–/GD+ families, local sequence changes in gene duplicates were estimated with a sliding window of 100 aa (see Materials and Methods). Similar to what we found above for AS+/GD+ sequence families (Figure 3B), for GD80 families the local seq.id. between gene duplicates without AS (AS–/GD+) is substantially higher than the local seq.id. between alternative splice variants without duplicates (AS+/GD–) (Figure 3D). For GD40 families, the overlap with AS data is larger; nonetheless, the overall trend is consistent with that shown in Figure 3C.

#### The nature of the sequence changes.

To complete our analysis of substitutions, we compare the nature of the amino acid replacements in AS and GD, focusing on nonconservative replacements. These involve amino acids of very different physico–chemical properties, and are thus more likely to alter protein structure, stability, and function [25,26] We find that, in general, the percentage of nonconservative amino acid replacements is higher for AS than for GD substitutions, a situation that holds whether we consider GD families at the 80% (58% and 44% nonconservative replacements for AS and GD, respectively) or 40% seq.id. level (60% and 43% nonconservative replacements for AS and GD, respectively). Thus, AS substitutions are physico–chemically more aggressive than GD substitutions.

**Figure 4:**
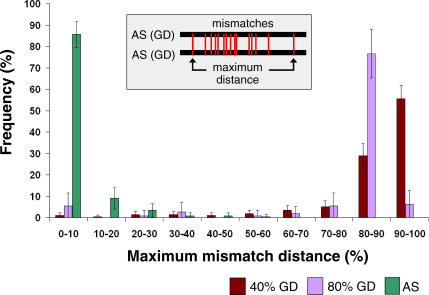
The Distribution of Nonconservative Changes along Sequences The maximal mismatch distance between nonconservative substitutions is much smaller in AS than in GD. The maximal mismatch distance is the number of residues between the two most distant, nonconservative substitutions, normalized by sequence length. Nonconservative mismatches have a negative value in the Blosum62 matrix [65] and were chosen for their stronger impact on protein structure and function. The plot depicts AS data in green, and GD data for families at 80% and 40% seq.id. in light violet and dark red, respectively. We observe that nonconservative substitutions in AS are much more localized than those in GD.

**Figure 5:**
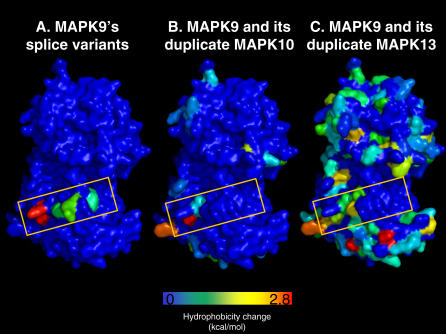
The 3-D Distribution of Physico–Chemical Changes in the Affected Residues of AS and GD The example of mitogen-activated protein kinase 9 (MAPK9). The example of human MAPK9 illustrates how differences between AS and GD in the distribution of sequence changes result in different distributions of physico–chemical properties across the 3-D structure. The original structure of MAPK9 was homology-modelled after MAPK10 and is shown in blue; the residue changes are indicated following a colour scale related to the associated difference in hydrophobicity (we use the absolute value of the difference in order to avoid too many colours; the colour scale goes from blue to red, where the latter corresponds to the largest change). For comparison purposes, the location of the AS changes in the three structures is indicated by a yellow box. As a hydrophobicity measure, we used the free energy of water to octanol transfer [77]. (A) Alternative splice isoforms of MAPK9. (B) Gene duplicates of high seq.id. (MAPK10; isoform alpha2, 84% seq.id. to MAPK9). (C) Gene duplicates of medium seq.id. (MAPK13; 46% seq.id. to MAPK9). We observe, in accordance with the results from the sequence analysis, that while AS changes are located at a very specific location, GD changes are spread all over the protein surface. As expected, the number of changes between MAPK9 and MAPK13 is the largest. Neither one of MAPK9′s paralogues (MAPK10 and MAPK13) shows a set of residue changes identical to that in the alternative splice variant.

Further, we use the maximal distance between replacements as a measure of the distribution of nonconservative changes along the sequence. We find clear differences between AS and GD (Figure 4), in accordance with the previous results. In particular, we observe that, for GD families at both the 40% and 80% levels, replacements are significantly more spread along the sequence than those for AS substitutions. This concentration of AS changes in the sequence in turn may result in a highly localized change in the 3-D distribution of physico–chemical properties, contrary to what happens with GD. Figure 5 illustrates this point using the example of MAPK9.

In summary, AS replacements are generally more concentrated and physico–chemically drastic than those observed for GD. The nature of these differences argue against interchangeability between AS and GD, on the basis of existing studies [23–26].

### Insertions and Deletions

#### Size.

**Figure 6:**
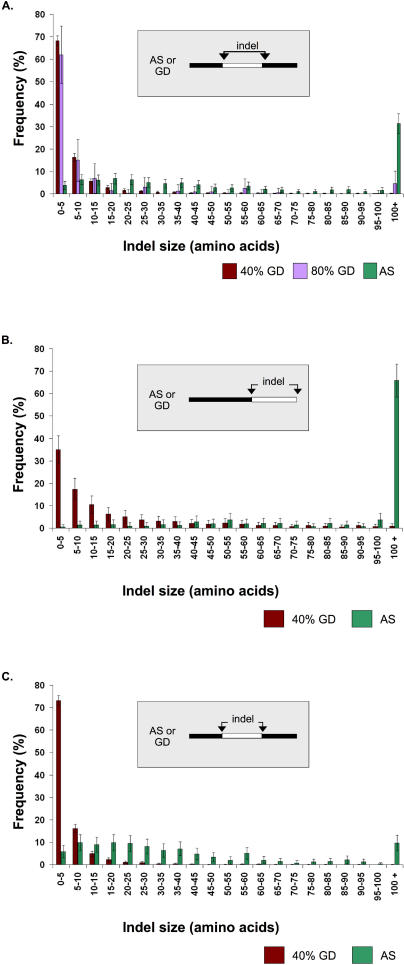
The Size Distribution of Insertions/Deletions in AS and GD All analyses of indels have been made for gene families with both AS and GD (i.e., AS+/GD+). (A) AS indels are longer than GD indels. Indels for GD were obtained from the alignments of GD families at 40% (dark red) and 80% (light violet) seq.id. Information on AS indels (green) was obtained from the SwissProt record of the corresponding protein. Indel size distributions for both GD40 and GD80 are very similar, with most of the indels being shorter than five residues. In contrast, many AS indels are longer than 100 residues. (B,C) Size distribution for external and internal indels in AS and GD. External indels (B) lie at the N- or C-terminal ends of the protein; internal indels (C) lie in the middle. AS and GD40 indel sizes are different depending on the position of the indels in the sequence. While AS indels are generally larger than GD indels (also see Figure 6A), external indels (B) are larger than internal ones (C), both for AS and GD. The shift in indel sizes implies that large indels (as often introduced by AS) are better-tolerated at the N- and C-termini of proteins, where they are less likely to induce important structural changes.

Second, we studied indels, which modify protein structure in a different way than substitutions. A first and intuitive measure of their impact is provided by indel size: small indels are more likely to have a small effect on structure than larger ones. We find that indel sizes are substantially different for AS and GD (Figure 6A) for both GD40 and GD80. AS indels are of domain size (≥30aa, many even >100aa) in agreement with previous results [27,28]. In contrast, about three-quarters of GD indels are fewer than five residues long, which means that they are shorter than a domain (Figure 6A). Thus, AS has a strong prevalence over GD for indels of whole domains. Changes in the domain composition, in turn, can modulate protein function very abruptly—for example, by on/off switch mechanisms that result in dominant-negative regulators [29,30].

#### Location of indels.

Given that, in general, sequence changes are severely constrained by structure and stability requirements [10], it seems difficult to rationalize how AS and GD indels can be so different. A detailed explanation can only come from the structure comparison of many pairs of alternative splice isoforms for which we still lack data [31–33]. Nonetheless, a reasonable approximation can be obtained following simple considerations. The N- and C-termini usually occur at the protein surface [34]. At the terminus, inserting or deleting a sequence stretch is likely to have less of an effect on the protein's structure than internal indels, and thus external indels may be less restrained in their size.

We separated the AS and GD indels according to their location in sequence (N-/C-terminal ends or internal) and plotted the corresponding size distributions (Figure 6B–6C). The very large AS indels (>100 aa) are usually located at the N- or C-terminal end where they are less likely to disrupt protein structure/function. Similarly, GD indels are generally larger at external than at internal positions, but the trend is much weaker than for AS. More importantly, internal AS indels tend to be larger than internal GD indels, indicating that they have a stronger potential to interfere with the structure of the protein core.

#### Overlap between indels in AS and GD.

**Figure 7:**
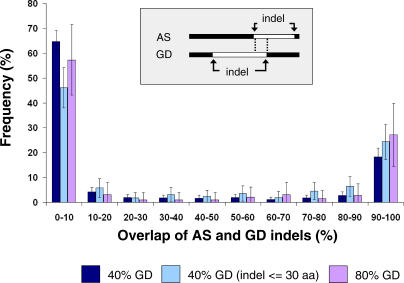
The Overlap between AS and GD Insertions/Deletions The overlap between AS and GD indels is very small. For the frequency distribution of the overlap between AS and GD indels, AS indels were taken as reference. GD data at 80% seq.id. are shown in light violet, while GD data at 40% seq.id. are shown in dark and light blue for both all indels and only short indels (≤30aa), respectively. Given the small overlap, AS and GD indels are likely to affect different locations in protein structure.

We also examined the overlap between the location of indels in splice variants and in duplicates of the same gene (Figure 7). Most AS indels (~80%) show no or negligible overlap with GD indels. The same holds true when focusing on very short indels of <30 aa length. Thus, AS indels affect different regions of protein structure, diversifying protein function in different ways from GD indels. As shown in Figure 7, short AS indels occur in sequence regions different from those affected by GD and thus are more likely to involve core residues, or relevant secondary structure elements. An example of such drastic effect of AS is the rat Piccolo C_2_A domain, in which splicing removes a nine-residue sequence stretch, which changes secondary structure and abrogates dimerization [35].

There is also a small fraction of instances (~15%) with an obvious overlap between AS and GD indels (Figure 7). Examined closely, we find that some of them could be instances of equivalent changes in AS and GD that support molecular and direct interchangeability. However, in contrast to examples such as that of U2AF35, which represents substitutions [9] (Figure 1A), the few equivalent changes in our dataset consisted of a simpler event, i.e., a deletion, producing a long and a short alternative splice isoform and a gene duplicate homologous to the short alternative splice isoform.

In summary, the results obtained in the study of indels lead to the same conclusions as for substitutions: in general, the impact of AS and GD on protein function is not interchangeable, irrespective of whether we consider GD at the 80% or 40% seq.id. levels.

### Conclusions and Possible Explanations of the Inverse Correlation between AS and GD

AS and GD are anticorrelated at the genomic level (Figure 2A) [12,13]. This relationship holds true for genes of different degrees of sequence divergence and of different functions, and suggests functional interchangeability between both phenomena [12]. This hypothesis is supported by known examples of equivalence [6–9] and by general, structural constraints on sequence changes [10]. In contrast, our sequence analysis shows that AS and GD followed by divergence are not directly interchangeable in their effect on protein structure and function. Indeed, they introduce very different changes with respect to indels and substitutions. The differences are summarised in Table 1. Indels caused by AS are more drastic than those observed in GD, both in type and location of affected residues. In the case of substitutions, we find that for GD they are more conservative and more broadly distributed along the whole sequence than those of AS. However, we also observe a small number of cases in which the interchangeability hypothesis may be true, and the sequence changes introduced by AS and GD are equivalent (Figure 3C, Figure 7).

**Table 1:**
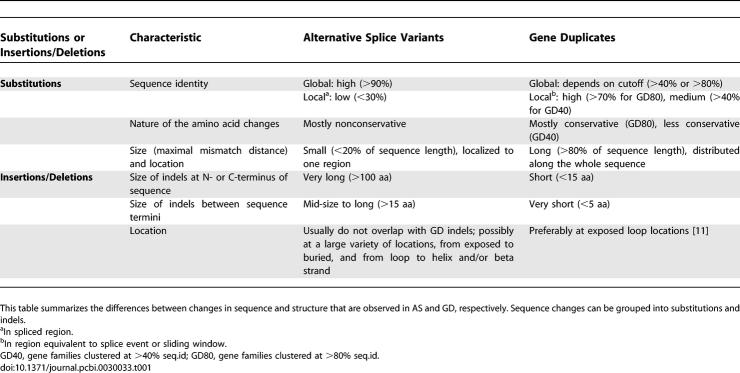
Summary of the Effects of Alternative Splicing and Gene Duplication on Sequence and Structure

To explain the apparent paradox between the relationship of AS and GD at the genome and at the protein level, we speculate on alternative explanations for the depletion of AS observed in large GD families (Figure 2A) [12,13], discussing several effects that may contribute to it. Duplicated genes tend to have fewer exons than genes that have no duplicates but alternative splice variants (see Figure S8). This trend may in part be due to retrotransposition events which create duplicates which are single-exon genes that cannot have splice variants. However, when accounting for single-exon genes, the anticorrelation between AS and GD is still observed (see Kopelman et al. [12] and Figure S11). In addition, the anticorrelation may arise from a combination of the negative selection pressures introduced by dosage balance effects. In singleton genes that have evolved a splice variant, duplication may be disfavoured due to a multiple simultaneous dosage balance effect [36]: a single duplication event would produce a multitude of additional gene variants whose functions interfere with the existing, tightly regulated system of biological processes. This effect would be weaker for genes without AS, thus introducing a bias towards their duplication. This bias may be reinforced, under conditions of environmental stress [37,38], by the need to increase expression levels of a desirable function [39,40]. Further, the retention of duplicates as a potential backup system may be fostered if a gene is essential for the cell's survival upon gene knockout [41]. In contrast, the introduction of AS in essential genes does not directly support robustness against null-mutations, and may even be slightly deleterious. Thus, essentiality of genes may contribute to the inverse relationship between AS and GD.

Finally, if a gene with AS has duplicated, subsequent loss of an isoform in one of the copies may be tolerated due to the existence of an identical version of this isoform in the other copy of the gene. This explanation is supported by recent findings on the evolution of AS upon GD [13], and the fact that the depletion in AS is stronger for closely related duplicates [12,13] (Figure 2A, 80% seq.id.). Such a scenario would also be an extension of the backup role shown for paralogues [42].

A combination of these effects would, in general, result in a smaller proportion of genes with AS in gene families with more than one duplicate, in particular for recent duplicates, suggesting that the chronological order of events plays a role. Subsequent divergence of the gene duplicates may alleviate the negative impact of the dosage balance effect, allowing the evolution of AS and reducing the anticorrelation between AS and GD.

## Materials and Methods

### Genomic Analysis

#### Datasets.

To test the relationship between GD and AS at the genomic level, we used: (i) clusters of homologous sequences inferred from the seq.id. (equivalent to gene families); (ii) sets of known or predicted alternative splice variants, or isoforms, for a particular gene. An overview of the data is provided in Table S1. For AS genes, we primarily used the datasets downloadable from EBI AltSplice server (releases 2.0 for both mouse and human) [43].

In addition, we compared the findings using the AltSplice dataset with findings using data from the SwissProt database [44] and the Ensembl predictions of splice variants [45] (see Table S1). Genes that have more than one transcript variant in either one of the three sets are denoted AS+. A small number of false positives can be expected in each of the three AS+ sets. Within the set of sequences without AS (AS−), we expect a fraction of false negatives, that is, genes that splice alternatively but for which we have no data yet. However, it is unlikely that these false positives or false negatives coincide across each dataset, as each database used its own approach to derive the data, and we expect no systematic error. All three datasets show the same trends (see Table S1), consistent with previous findings [12,13].

To estimate GD, we used the Ensembl protein predictions for human (version 37.35j) and mouse (version 37.34e) (Table S1). Both genomes were made nonredundant in that we only included the longest, predicted transcript for each gene, and the sequences were filtered for low-complexity regions using the seg program [46]. To obtain families of paralogous genes, the sequences were clustered to 40%, 60%, 80%, and 90% seq.id. using CD-HIT [47]. The higher the seq.id. cutoff for a family, the more conserved are the family members: clusters of 40% seq.id. are expected to be larger and contain more distantly related sequences than clusters of 80% seqid. Clusters with more than one sequence are termed GD*+*, denoting the existence of homologues. Figure S1 shows the distribution of human sequences across GD families of different sizes, using a variety of seq.id. cutoffs. The human–yeast, human–fly, and human–mouse orthologues were derived from the InParanoid database [48].

Retrotransposition creates gene duplicates that have only a single exon, and hence are unlikely to show any AS. To test for a possible bias in GD families (GD+) stemming from retrotransposition, we examined the distribution of single-exon genes across AS and GD sets using the SEGE database [49], similar to an approach described by Kopelman and colleagues [12]. The procedure followed was: (i) all human genes were clustered according to 80% seq.id.; (ii) all genes were labelled according to their known AS; and (iii) the number of single-exon genes [49] amongst singletons, gene families, and genes with/without AS was calculated. This distribution across genes with AS and/or GD is shown in Table S2. If retrotransposition was a major source of GD without AS (AS−/GD+), then we would expect to see a bias towards single-exon genes in this category. This is not the case; also see Kopelman et al. [12].

#### Function analysis.

To determine whether the inverse relationship between AS and GD is specific to genes of particular functions, we analyzed functions for human proteins of the four different sets of genes with or without AS or GD (AS+/GD+, AS+/GD−, AS−/GD+, AS−/GD−). We analyzed gene functions from both the SwissProt and the AltSplice database using the DAVID Web server (http://david.abcc.ncifcrf.gov/home.jsp) [50]. DAVID analyzes human proteins for biases in terms of Gene Ontology [51] terms, protein domains, pathways, functional categories, protein interactions, disease, literature, and general annotations (sequence features). As a background list for comparisons, we used the union of the four sets described above. The detailed results are listed in Table S3.

#### Expression analysis.

In this part of our work, we compared the extent of coexpression between alternative splice isoforms (AS coexpression) with that of coexpression between gene duplicates (GD coexpression). To estimate AS coexpression across human genes, we analyzed data on absolute expression levels of exon junctions of 3,840 human genes, measured across 44 different tissues [17] (Geo [52] GDS829–GDS834). AS coexpression of a gene was measured as the average pairwise PC between the expression vectors of all its exon junctions. A high PC indicates low coexpression (low variation) amongst the exon junctions and hence splice variants, and vice versa.

The expression of all exon junctions of a particular gene was then summarised (averaged) to form one vector representing the overall expression pattern of a gene. We measured GD coexpression of a GD family, i.e., the amount of coexpression amongst gene duplicates, as the average pairwise PC between the gene expression vectors of all family members. For gene families of >80% seq.id., the 3,840 genes in the dataset [17] did not provide enough families with more than two members to be suitable for further analysis. Thus, we examined another dataset with absolute expression values of human genes across 79 different tissues published by Su et al. [53] (Geo [52], GDS596). In contrast to the first dataset, GDS596 reports expression per gene and not per exon junction, and thus is only suited for analysis of GD coexpression, not AS coexpression. AS+ genes were defined as given by the AltSplice database [43]. Please refer to Table S4 for analysis of a third dataset, use of other measures of coexpression, and different post-processing procedures of the expression data.

### Protein Sequence Analysis

#### Datasets.

We assessed GD at the whole-gene level in which two proteins are assigned to the same gene family when their seq.id. is above 40%, or above 80% (GD40 or GD80, respectively). These families were obtained by clustering the SwissProt [44] human proteins using the program CD-HIT [47] (http://bioinformatics.burnham-inst.org/cd-hit). We also used the Pfam [54] domain families as a model of highly diverged gene families. More precisely, we used all the Pfam [54] families that mapped to one or several of the SwissProt [44] proteins in the AS dataset. The results obtained with this model are shown in Figure S5. To allow proper testing of the interchangeability, we focused on AS+/GD+ families, i.e., those families for which at least one gene duplicate and one splice variant are known.

Our set of genes with AS was obtained after querying the SwissProt database [44] version 40, with the keywords VARSPLIC and HUMAN, or MOUSE, respectively. A summary of the number of genes, isoforms, and duplicates in the datasets used in this work is given in Table S5.

SwissProt [44] is a high-quality, manually curated database that has been recently used by different research groups in the study of AS at the protein level [20,27,28,31,55–57]. While the data may be biased by the curation process, several facts suggest that this potential bias would not affect our results. First, the proportions of isoforms showing indels and substitutions in our sample, 73% and 27%, respectively, are comparable with those inferred from other studies: 76% and 24% [58], and 67% and 33% [59]. Second, the anticorrelation observed between AS and GD [12,13] (Figure 2A) can be reproduced with SwissProt data [44] (Figure S11). Third, our data could be biased by the existence of nonfunctional isoforms targeted for nonsense-mediated mRNA decay (NMD) [60]. When repeating our analysis after eliminating those isoforms that can be targeted to NMD (about 8% of the data [61]), there was no difference in the results compared with those from the original data (see Figure S6A). Finally, for AS indels, results obtained from SwissProt [44] are comparable with those from another AS database, ASAP [62] (Figure S7). Therefore, we do not expect a significant bias in our results when using SwissProt AS data.

For all of the distributions shown in the different figures, we computed the confidence interval corresponding to each proportion in the distribution, following Goodman [63].

#### Characterization of substitutions.

Our work involves detailed comparison between AS and GD sequence changes which occur between alternative splice isoforms or between gene duplicates (Figure 1B and 1C).

*Global and local seq.id.* Global seq.id. corresponds to the commonly used percentage of seq.id. between aligned sequences. It was computed from a whole-sequence alignment between the sequences of either two duplicates or two alternative splice isoforms, using standard dynamic programming [64]. When comparing alternative splice isoforms, one of the sequences was always that of the SwissProt [44] reference isoform.

Local seq.id. refers to the seq.id. between parts of the sequences. Local seq.id. between alternative splice isoforms was always computed in the same way, comparing the sequence stretches substituted between them. To this end, we first obtained the location of these stretches from SwissProt [44], and then we aligned them using a standard dynamic programming method [64]; the local seq.id. was computed from this alignment. To avoid meaningless comparisons, we introduced some restrictions [56]: (i) both sequence stretches must be >10 aa, and (ii) the size of the shorter stretch must be at least 60% that of the larger stretch. These filters were only applied when computing the local seq.id. but for no other variable.

To obtain local seq.id. between gene duplicates, we distinguished two cases (Figure S9): either we observe both AS and GD for a given gene (AS+/GD+), or we only observe GD but not AS (AS–/GD+).

In AS+/GD+ cases, we followed two different procedures. The first one uses a sliding window of the size of the AS substitution, *N* (Figure S9A). For each gene, we then (i) aligned the sequence of the SwissProt reference sequence for the gene (usually that of the longer isoform) with that of one of the gene duplicates; (ii) computed the identity percentage between positions *i* and *i + N − 1*, at all possible *i* locations of the window along the alignment; and (iii) repeated steps (i) and (ii) for each comparison between the first gene and any of its duplicates. *N,* the size of the window, and the AS substitution were obtained from SwissProt annotations [44] (Figure 3B).

In the second procedure analysing the AS+/GD+ case (Figure S9B), we studied interchangeability of sequence changes at the AS location. To this end, we first aligned the sequence of the protein with known splicing to one of its duplicates. The former was always the sequence of the SwissProt [44] reference isoform. Then, we mapped location and length of the AS substituted stretch to the sequence of the gene duplicate and computed seq.id. between both sequence stretches. The information on the location and length of the AS substituted stretch was obtained from the SwissProt [44] annotations (Figure 3C).

In AS−/GD+ cases, a direct comparison between AS and GD local seq.id. is no longer possible. Here, local seq.id. was estimated using a moving window of size *N*: (i) we aligned the sequences of the two duplicates, and (ii) we computed the identity percentage between the positions *i* and *I + N – 1*, at all possible locations *i* of the moving window along the alignment. We calculated these local seq.id. for GD using *N* = 100 aa; the resulting distribution is shown in Figure 3D.

*Nonconservative changes.* We define nonconservative changes as those for which the corresponding value of the Blosum62 [65] substitution matrix is negative. This criterion has been used in the annotation of SNPs [66]. The fraction of nonconservative mismatches between two sequences was obtained by dividing the number of mismatches with a negative Blosum62 value by the total number of mismatches in the alignment. The percentages of nonconservative mismatches for AS and GD substitutions were compared using the T-test (http://home.clara.net/sisa).

*Distribution of the maximal distance between nonconservative mismatches.* The maximal distance between mismatches corresponds to the sequence separation between the two most distant mismatches in a sequence alignment. In the case of GD, we (i) aligned all the sequences of a given gene family with the reference sequence of the member (or members) that has AS; (ii) for each alignment, mapped the mismatches to the sequence which is the reference isoform in SwissProt; (iii) for each alignment, computed the distance between the two most separated mismatches as follows: *(j_r_ – i_r_)/N_r_*, where *j_r_* and *i_r_* are the sequence locations of the closest mismatches to the C- and N- termini, respectively; *N_r_* is the size of the latter; and (iv) repeated steps (ii)–(iii) for all the alignments obtained in (i) and binned the resulting values.

In the case of AS, the maximal distance between mismatches was computed using the same equation as before: *(j_r_ – i_r_)/N_r_*, where in this case *j_r_* and *i_r_* correspond to the locations of the end and beginning of the substituted fragment, as provided by SwissProt.

Note that the maximal mismatch distance was normalized by the sequence length to allow comparison of all results independent of the protein size.

*Comparative modeling.* The structure of mitogen-activated protein kinase 9 was modelled using that of mitogen-activated protein kinase 10 [67]. The seq.id. between both proteins is 84%, which guarantees a good modelling result. The alignment between the two protein sequences was generated employing standard dynamic programming [64]. The resulting alignment was used as input to run the comparative modelling program MODELLER [68].

#### Characterization of indels.

For all the variables, we conducted a comparison between AS and GD as described before (Figure 1B–1C).

*Indel sizes.* The indel size distribution for the AS events was obtained from the SwissProt [44] records of the proteins in our dataset. The procedure was: (i) records with VARSPLIC annotations were parsed for the presence of MISSING annotations; (ii) the MISSING annotation provided the initial (M) and final (N) positions of the inserted/deleted fragment; and (iii) the length of the indel was computed as *N − M + 1*. The resulting lengths were binned to give the size distribution shown in Figure 6A.

In the case of the indel size distribution for GD the procedure was: (i) for each gene family in our dataset (see above) we obtained the length of all indels (gaps) for all the possible alignments between the proteins in the family, and (ii) the resulting lengths were binned after a simple redundancy correction. The redundancy correction consisted of dividing the contribution of each indel in the frequency histogram by the number of sequences in the family cluster. The resulting distribution follows a power law very similar to that previously found by Benner and colleagues in a massive alignment experiment [69], supporting the reliability of our data.

Both the AS and GD indels datasets were subsequently broken down in two subsets, according to whether indels were external (positioned at the N- or C-terminal ends of the protein sequence) or internal (positioned within the protein sequence). The resulting length distributions are shown in Figure 6B–6C.

*Overlap between indels*. The procedure to estimate overlaps between AS and GD indels was: (i) map the AS indel to the sequence of the longest isoform; (ii) align the sequence of that isoform to that of the other genes in the family; (iii) map the indels from the previous alignments to the longest isoform; (iv) for each possible comparison between the AS indel and one GD indel compute the amount of common amino acids and divide it by the size of the AS indel; and (v) bin the results after redundancy correction. The redundancy correction consisted of only adding one count to the frequency histogram when the overlaps between a given AS indel and a series of GD indels were always the same.

As mentioned before, the SwissProt database [44] is of high quality but small; thus, we expect to have missed a certain number of alternative splice isoforms, which are likely to increase the number of cases with high overlap between AS and GD indels.

## Supporting Information

Figure S1 Distribution of GD Family Sizes Distribution of human sequences across GD families as determined by different seq.id. cutoffs (40%, 60%, 80%, 90%). GD families of size 1 denote singletons, i.e., genes without paralogues (GD−).
(54 KB PPT)

Figure S2 The Distribution of Molecular Function and Biological Process We tested for functional biases across proteins with AS and/or GD using the GO annotation available for humans from the GO database [70]. For a more detailed analysis of function characteristics, see Table S3.
Human genes were annotated with respect to biological process (A) and molecular function (B) using GO annotation [51,70]. GD families were determined according to an 80% seq.id. cutoff; AS family information was taken from the AltSplice database [43]. All sequences were assigned to one of the four sets, and the distribution of biological processes (A) and molecular functions (B) is shown for the four sets separately: AS−/GD− no duplication or AS known; AS−/GD+ duplicates, but no AS known; AS+/GD− no duplication, but AS known; and AS+/GD+ both duplicates and alternative splice variants known. There are no obvious biases in the function composition for any of the four constellations of AS/GD.
(749 KB PPT)

Figure S3 Chromosomal Location of the Duplicated Genes We show the fraction of duplicated genes per gene family that have different chromosomal location, using a 40% seq.id. cutoff (dark red). (Data for GD80 families are not shown because of the small amount of data.) In all except one group of families, on average >55% genes within a family have different chromosomal locations. This indicates different regulation between duplicates [71] and therefore no interchangeability between AS and GD, given that transcription and mRNA splicing are tightly coupled [72,73].
(55 KB PPT)

Figure S4 Analysis for Mouse (40% seq.id. Cutoff) The four figures reproduce, for mouse, the analysis shown in Figures 3A, 4, 6A, and 7, respectively The results are qualitatively identical to those discussed for human in the main text, and support the idea that in general AS and GD are not interchangeable at the molecular level. Due to the smaller amount of data, the analysis at 80% cutoff did not produce statistically meaningful results.
(A) Substitutions in AS have different effects on global versus local seq.id. Light and dark green correspond to global and local seq.id. for AS substitutions, respectively. Global seq.id. is obtained after aligning two isoforms for the same gene, for which the AS event involved a substitution. Local identity applies only to the substituted stretches. Dark red corresponds to the seq.id. distribution for GD families at 40%, after sequence alignment between paralogues. The global seq.id. between splice isoforms is very high while the local seq.id. in alternative splicing variants is very low. Both seq.id. distributions for AS contrast with those of GD families.
(B) Maximal mismatch distance between nonconservative substitutions is much smaller in AS than in GD. The maximal mismatch distance is the number of residues between the two most distant, nonconservative substitutions, normalized by whole sequence length. Nonconservative mismatches have a negative value in the Blosum62 matrix and were chosen for their stronger impact in protein structure and function. The plot depicts AS data in green, and GD data for families at 40% seq.id. in dark red. Substitutions in alternative splice variants are much more localized than those in gene duplicates.
(C) Size distribution for indels. The AS distribution is shown in green. Indels for GD are shown for the whole-gene model (dark red). Clear differences are found between both distributions.
(D) Frequency distribution of the amount of overlap between AS and GD indels, taking as reference the sequence of the AS indel (see Materials and Methods). Dark blue bars correspond to the case when indels of any size are considered. Light blue bars correspond to the case when only subdomain indels (≤30 aa) are considered.
(1.1 MB PPT)

Figure S5 Comparison between AS, Whole-Gene, and Domain-Based GD Families To provide another definition of gene families, we estimated GD families based on domain families. We used domain annotations from the Pfam database [54] that mapped to one or several of the SwissProt [44] proteins in the AS dataset. Nonhuman sequences were removed from the alignment.
(A) Global seq.id. distribution. The distribution of human AS sequences is shown in green; for GD whole-gene families (40% level) are shown in dark red; indel sizes for GD families defined by Pfam domains are shown in light red. We observe that the range of seq.id. for the latter is much lower than for AS and GD whole-gene families. At the local level (results not shown) the range of seq.id. for the Pfam model of GD is lower than that observed for AS. However, for the former the amino acid replacements spread over the whole sequence, contrary to what we observe for AS.
(B) The indel size distribution of human AS sequences is shown in green. Indel sizes for GD whole-gene families (seq.id. cutoff of 40%) are shown in dark red; indel sizes for GD families defined by Pfam domains are shown in light red. In the former, whole sequences were compared within each family to obtain the indel size distribution. In the domain-based GD families, indels were obtained from the multiple sequence alignments of the Pfam databank [54] Indels for both GD models show behaviour similar to that described by Benner and colleagues [69].
(C,D) Size distributions for external and internal indels, respectively, with the same colour code as in (B). These distributions indicate that indels from Pfam domains and GD families show similar trends when compared with AS indels. Overall, our results indicate that GD and AS are in general different in their sequence/structure changes, independently of the model representing GD.
(1.1 MB PPT)

Figure S6 Effect of Filtering Out Putative NMD Targets from the AS Data No significant differences are found between the original results and those obtained after eliminating from the AS dataset all the isoforms that may be targets of NMD machinery [61].
(A) Overall versus local seq.id. Original AS global and local seq.id. are shown in light and dark green, respectively. Overall and local seq.id. for NMD-filtered AS are shown in orange and yellow, respectively.
(B) Maximal mismatch distance between nonconservative substitutions. Original AS data are shown in dark green, NMD-filtered data are shown in orange.
(C) Indel size. Original AS data are shown in dark green, NMD-filtered data are shown in orange.
(D) Overlap between AS and GD indels. Original data are shown in violet, dark blue, and light blue, while the corresponding NMD-filtered data are shown in yellow, orange, and light green.
(1.3 MB PPT)

Figure S7 Excluding Potential Database Biases To exclude biases in our results introduced by the use of the SwissProt database [44] (dark green), we compared some of the findings with those obtained from using the ASAP database [62] (dark violet). The data are for human. Here we show the indel size distribution obtained using data from both databases. No obvious differences are found between the SwissProt and ASAP distributions that may affect the validity of our results.
(35 KB PPT)

Figure S8 Number of Exons per Gene To obtain the number of exons per gene, we followed the procedure employed by Saxonov and colleagues to build the EID database [74]. For each sequence, we obtain the exon information from the corresponding NCBI's GenBank [75], looking at the CDS join feature.
Three distributions show the number of exons per gene, corresponding to the following cases: singleton genes with AS (AS+/GD−, dark green); genes that are both duplicated and have AS (AS+/GD+, light green), and duplicated genes with no AS (AS−/GD+, dark blue). The results are obtained for gene families at both the 80% level (A) and the 40% level (B). In both cases we see that there is a trend for AS−/GD+ to have a smaller number of exons than AS+/GD+ and AS+/GD− genes.
(525 KB PPT)

Figure S9 Computation of the Local Sequence Identity Between Gene Duplicates We describe the two procedures followed to compute the local seq.id. between duplicates (see Materials and Methods).
(A) The first procedure is based on the use of a moving window the size, N, of the AS event. The window is moved along the aligned sequences of both duplicates, and at each position the seq.id. between them is computed (within the limits of the window).
(B) In the second procedure, we first aligned the sequence of the protein with known splicing to one of its duplicates. The former was always the sequence of the SwissProt [44] reference isoform. Then, we mapped location and length of the AS substituted stretch to the sequence of the duplicate and computed seq.id. between both sequence stretches.
(462 KB PPT)

Figure S10 Overview of the Expression Data Analysis (A) Illustrates the basic comparisons of coexpression, whose results are shown in Figure 2C and 2D. In Figure 2C, we compare coexpression amongst gene duplicates (GD coexpression) of GD families with and without alternative splice variants. Data on expression of gene duplicates come from two “conventional” datasets [53,76] (GDS596 and GDS1096) and the data from Johnson et al. (GDS829–834; (B)) [17]. Data on the existence of alternative splice variants is from the AltSplice database [43]. In Figure 2D, we compare coexpression amongst exon junctions [17], approximating the extent of AS (AS coexpression) of GD− families and singleton genes.
(B) In the datasets published by Johnson et al. [17], each of the 3,840 human genes is represented by a matrix of absolute expression values of all exon junctions across 44 different tissues. We estimate AS coexpression by analyzing the variation of expression values in each gene's matrix. The average expression value of all exon junctions across the different tissues forms a vector representing the gene's overall expression pattern.
For each gene family, we can produce a second matrix of gene expression patterns of the duplicates across different tissues. We estimate GD coexpression by analyzing the variation of expression values in each gene family's matrix. GD coexpression was analyzed for the dataset by Johnson et al. [17] and two conventional [53] gene expression datasets (see Table S4).
We tested the following measures for analysis of coexpression. (i) The average pairwise PC. We calculated average PC between each pair of row vectors in the AS or GD matrix. PC close to 0 indicates no correlation in expression between exon junctions (representing AS) or gene duplicates, respectively. PC close to 1 indicates strong correlation between the row vectors and is indicative of little AS or differential expression amongst gene duplicates. (ii) The number of *unique* binarized row vectors per matrix. To normalize for the number of exon junctions per gene or number of gene duplicates in a family, we divided the number of unique row vectors by the total number of row vectors per matrix. We also tested relative entropy *RE* as a measure of coexpression. We calculated the relative entropy *RE* (mutual information) for each AS or GD matrix as the sum of p_obs_*log_2_(*p_obs_/p_exp_*) calculated for each column, where *p_obs_* is the observed frequency of the exon junctions or gene duplicates in one column and *p_exp_* is the expected frequency of all exon junctions or gene duplicates across all experiments. However, relative entropy did not prove to be a useful measure of matrix variation in our case, as it did not capture differential expression patterns (row vectors) but only general entropy in the matrix.
While matrices in the figure show binary expression data, calculations were done on both raw and binary data. All results are similar irrespective of the cutoff for binarization (600 or 150). They are also similar irrespective of the cutoff for gene family definitions (40%, 60%, or 80% seq.id.) or of the underlying AS+ datasets employed (SwissProt or AltSplice).
(746 KB PPT)

Figure S11 Anticorrelation between Family Size and Percentage of Genes with AS An anticorrelation between AS and GD [12,13] can also be produced using SwissProt [44] data.
(66 KB PPT)

Table S1 Genomic Data Overview Provides an overview of the genomic data from the Ensembl database (human release 37.35j, mouse release 37.34e) [1] and the AS data from the AltSplice database (release 2.0 for human and mouse AS) [2], SwissProt [3], and from the Ensembl annotations.
(54 KB DOC)

Table S2 The Distribution of Single-Exon Genes across Human Sequences Retrotransposition produces duplicates that consist of only one exon. To test for possible bias in families of gene duplicates (GD+) stemming from retrotransposition, we examined the distribution of single-exon genes across AS and GD sets using the SEGE database [1], similar to an approach described by Kopelman and colleagues [2]. The procedure followed was: (i) all human genes were clustered according to 80% seq.id.; (ii) all genes were labelled according to their known AS; and (iii) the number of single-exon genes [1] amongst singletons, gene families, and genes with/without AS was calculated.
(53 KB DOC)

Table S3 Function Analysis The table lists a selection of functions as obtained from the DAVID Web server [1], for the four protein sets (AS+/GD+, AS+/GD−, AS−/GD+, AS−/GD−) derived from SwissProt (A) and AltSplice (B), using an 80% seq.id. threshold to estimate GD. A more general overview of GO functions and biological processes across the datasets is shown in Figure S2.
All function annotations are significantly different from the background (E-value < 10^−10^). We removed redundant annotations and annotations that were too broad to be meaningful (e.g., “binding”). Duplication of particular gene families that are depleted in AS, such as ribosomal proteins or some receptors, has contributed to the inverse relationship between AS and GD, but cannot explain it completely.
(96 KB DOC)

Table S4 Analysis of Expression Data (375 KB DOC)

Table S5 Overview of the Dataset Employed in the Protein Sequence/Structure Analysis The table shows the number of genes with AS, and the number of multiple gene families, together with the respective number of sequences. Information on AS was taken from SwissProt [1]. The data are provided for human and mouse, for 40% and 80% seq.id. clusters.
(50 KB DOC)

### Accession Numbers

The accession numbers used in this paper are from Swiss-Prot (http://www.ebi.ac.uk/swissprot): rat Piccolo C_2_A Q9JKS6), human MAPK9 (P45984), MAPK10 (P53779), and MAPK13 (O15264); and from the Protein Databank (http://www.rcsb.org/pdb): MAPK10 (1jnk).

## Abbreviations

aa: amino acid(s)
AS: alternative splicing
AS−: gene without known splice variants
AS+: gene with splice variants
GD: gene duplication
GD−: gene without duplicates (as inferred from lacking sequence similarity to other sequences)
GD+: gene with duplicates (inferred from sequence similarity to other sequences)
GD40: gene families clustered at >40% sequence identitity
GD80: gene families clustered at >80% sequence identity
indel: insertion/deletion
NMD: nonsense-mediated decay
PC: Pearson correlation coefficient
seq.id.: sequence identity
