# Supplementary material for: The (In)dependence of Alternative Splicing and Gene Duplication
Source: PLoS Comput Biol. 2007 Mar 2;3(3):e33. doi: 10.1371/journal.pcbi.0030033 (PMC1808492; doi:10.1371/journal.pcbi.0030033)
Supplement: Supplementary file 1 [file pcbi.0030033.st001.doc]

## Table S1. Genomic data – overview.

The table provides an overview of the genomic data from the Ensembl database (human release 37.35j, mouse release 37.34e) [1] and the AS data from the AltSplice database (release 2.0 for human and mouse alternative splice variants) [2], SwissProt [3] and from the Ensembl annotations.

|  | **Human** | **Number of singletons (GD-) of these** | **Mouse** | **Number of singletons (GD-) of these** |
| --- | --- | --- | --- | --- |
| **Total number of genes** | 22176 | 40%: 12339  60%; 16314  80%: 18930 | 25419 | 40%: 13135  60%: 17603  80%: 20862 |
| **Number of AS+ genes from different databases** |  |  |  |  |
| Swissprot | 1026 | 40%: 571  60%: 788  80%: 931 |  |  |
| Altsplice | 9945 | 40%: 6516  60%: 8404  80%: 9377 | 8003 | 40%: 5200  60%: 6801  80%: 7613 |
| Ensembl | 7224 | 40%: 4172  60%: 5591  80%: 6455 | 4805 | 40%: 2706  60%: 3623  80%: 4241 |

**REFERENCES**

1. Birney E, Andrews D, Caccamo M, Chen Y, Clarke L, et al. (2006) Ensembl 2006. Nucleic Acids Res 34: D556-561.

2. Thanaraj TA, Stamm S, Clark F, Riethoven JJ, Le Texier V, et al. (2004) ASD: the Alternative Splicing Database. Nucleic Acids Res 32: D64-69.

3. Bairoch A, Apweiler R (2000) The SWISS-PROT protein sequence database and its supplement TrEMBL in 2000. Nucleic Acids Res 28: 45-48.
