# Supplementary material for: The (In)dependence of Alternative Splicing and Gene Duplication
Source: PLoS Comput Biol. 2007 Mar 2;3(3):e33. doi: 10.1371/journal.pcbi.0030033 (PMC1808492; doi:10.1371/journal.pcbi.0030033)
Supplement: Supplementary file 2 [file pcbi.0030033.st002.doc]

## Table S2. The distribution of single-exon genes across human

## sequences

Retrotransposition produces gene duplicates that consist of only one exon. In order to test for a possible bias in families of gene duplicates (GD+) stemming from retrotransposition, we examined the distribution of single-exon genes across AS and GD sets using the SEGE database[1], similar to an approach described by Kopelman and colleagues [2]. The procedure followed was: (i) all human genes were clustered according to 80% sequence identity; (ii) all genes were labelled according to their known AS; (iii) the number of single-exon genes [1] amongst singletons, gene families, and genes with/without AS was calculated.

|  | GD-  (Singletons) |  | GD+  (gene families) |  |
| --- | --- | --- | --- | --- |
|  | Single-exon genes | Total | Single-exon genes | Total |
| AS- | 106 (0.9%) | 11841 | 44 (1.6%) | 2824 |
| AS+ | 49 (0.7%) | 6912 | 33 (4.9%) | 672 |

**REFERENCES**

1. Sakharkar MK, Kangueane P (2004) Genome SEGE: a database for 'intronless' genes in eukaryotic genomes. BMC Bioinformatics 5: 67.

2. Kopelman NM, Lancet D, Yanai I (2005) Alternative splicing and gene duplication are inversely correlated evolutionary mechanisms. Nat Genet 37: 588-589.
