# Supplementary material for: The (In)dependence of Alternative Splicing and Gene Duplication
Source: PLoS Comput Biol. 2007 Mar 2;3(3):e33. doi: 10.1371/journal.pcbi.0030033 (PMC1808492; doi:10.1371/journal.pcbi.0030033)
Supplement: Supplementary file 3 [file pcbi.0030033.st003.doc]

## Table S3. Function analysis

The table lists a selection of functions as obtained from the DAVID webserver [1], for the four protein sets (AS+/GD+, AS+/GD-, AS-/GD+, AS-/GD+) derived from SwissProt (**A.**) and AltSplice (**B.**), using an 80% sequence identity threshold to estimate gene duplicates. A more general overview about GO functions and biological processes across the datasets is shown in **Figure S2**.

In the table, all function annotations are significantly different from the background (E-value < 10^-10^). We removed redundant annotations and annotations which were too broad to be meaningful (e.g. ‘binding’). Duplication of particular gene families which are depleted in alternative splicing, such as ribosomal proteins or some receptors, has contributed to the inverse relationship between AS and GD, but cannot explain it completely.

##### A. SwissProt, GD80

| **Type of annotation** | **Annotation** | **Number of proteins** | **% of list** | **E-value** |
| --- | --- | --- | --- | --- |
| **Gene set** |  |  |  |  |
| **AS-/GD-** |  |  |  |  |
|  | n/a |  |  |  |
| **AS-/GD+** |  |  |  |  |
| KEGG_PATHWAY | ribosome | 92 | 3.60% | 8.50E-62 |
| GOTERM_MF_ALL | structural constituent of ribosome | 119 | 4.60% | 3.40E-56 |
| SP_PIR_KEYWORDS | ribonucleoprotein | 82 | 3.20% | 6.60E-30 |
| SP_PIR_KEYWORDS | protein biosynthesis | 69 | 2.70% | 1.20E-28 |
| SP_PIR_KEYWORDS | nucleosome core | 29 | 1.10% | 2.00E-24 |
| INTERPRO_NAME | Histone core | 30 | 1.20% | 2.40E-24 |
| SP_PIR_KEYWORDS | GTP binding | 42 | 1.60% | 3.40E-23 |
| INTERPRO_NAME | Histone-fold/TFIID-TAF/NF-Y | 33 | 1.30% | 2.80E-22 |
| INTERPRO_NAME | Olfactory receptor | 71 | 2.70% | 1.10E-14 |
| SP_PIR_KEYWORDS | methylation | 30 | 1.20% | 1.50E-14 |
| GOTERM_CC_ALL | chromatin | 45 | 1.70% | 4.50E-14 |
| **AS+/GD-** |  |  |  |  |
| SP_PIR_KEYWORDS | phosphorylation | 231 | 23.40% | 3.20E-22 |
| SP_PIR_KEYWORDS | disease mutation | 157 | 15.90% | 4.10E-20 |
| GOTERM_BP_ALL | development | 207 | 21.00% | 4.70E-16 |
| **AS+/GD+** |  |  |  |  |
|  | n/a |  |  |  |

##### B. AltSplice, GD80

| **Type of annotation** | **Annotation** | **Number of proteins** | **% of list** | **E-value** |
| --- | --- | --- | --- | --- |
| **Gene set** |  |  |  |  |
| **AS-/GD-** |  |  |  |  |
| GOTERM_MF_ALL | rhodopsin-like receptor activity | 476 | 6.10% | 3.10E-81 |
| UP_SEQ_FEATURE | glycosylation site:N-linked (GlcNAc...) | 1460 | 18.60% | 1.70E-56 |
| SP_PIR_KEYWORDS | glycoprotein | 1493 | 19.00% | 2.60E-49 |
| SP_PIR_KEYWORDS | sensory transduction | 312 | 4.00% | 6.20E-35 |
| UP_SEQ_FEATURE | DNA-binding region: Homeobox | 119 | 1.50% | 2.70E-24 |
| **AS-/GD+** |  |  |  |  |
| INTERPRO_NAME | Olfactory receptor | 74 | 3.60% | 8.40E-26 |
| GOTERM_MF_ALL | structural constituent of ribosome | 69 | 3.40% | 7.10E-25 |
| INTERPRO_NAME | Histone core | 26 | 1.30% | 1.70E-22 |
| SP_PIR_KEYWORDS | nucleosome core | 25 | 1.20% | 3.40E-22 |
| GOTERM_BP_ALL | sensory perception of smell | 66 | 3.20% | 3.40E-19 |
| INTERPRO_NAME | Rhodopsin-like GPCR superfamily | 90 | 4.40% | 2.80E-16 |
| GOTERM_BP_ALL | chromatin assembly or disassembly | 36 | 1.80% | 2.00E-13 |
| UP_SEQ_FEATURE | cross-link:Glycyl lysine isopeptide (Lys-Gly) (interchain with G-Cter in ubiquitin) | 14 | 0.70% | 1.60E-12 |
| **AS+/GD-** |  |  |  |  |
| GOTERM_BP_ALL | biopolymer metabolism | 1518 | 15.80% | 4.70E-16 |
| GOTERM_BP_ALL | macromolecule metabolism | 2269 | 23.60% | 1.40E-12 |
| **AS+/GD+** |  |  |  |  |
| SP_PIR_KEYWORDS | ribosome | 36 | 4.90% | 1.10E-32 |
| SP_PIR_KEYWORDS | ribonucleoprotein | 53 | 7.20% | 4.60E-28 |
| SP_PIR_KEYWORDS | protein biosynthesis | 45 | 6.10% | 3.60E-26 |
| SP_PIR_KEYWORDS | GTP binding | 26 | 3.60% | 9.20E-18 |

##### REFERENCES

1. Dennis G, Jr., Sherman BT, Hosack DA, Yang J, Gao W, et al. (2003) DAVID: Database for Annotation, Visualization, and Integrated Discovery. Genome Biol 4: P3.
