# Supplementary material for: The (In)dependence of Alternative Splicing and Gene Duplication
Source: PLoS Comput Biol. 2007 Mar 2;3(3):e33. doi: 10.1371/journal.pcbi.0030033 (PMC1808492; doi:10.1371/journal.pcbi.0030033)
Supplement: Supplementary file 4 [file pcbi.0030033.st004.doc]

## Table S4. Analysis of expression data.

As mentioned in the main body of the article, we compared AS and GD in their effect on gene expression. In particular, we compared the degree of co-expression of AS isoforms (AS co-expression) with that of gene duplicates (GD co-expression). For the basic protocol, see **Figure S10**. To estimate AS co-expression across subsets of human genes, we analyzed data on absolute expression levels of exon-junctions of 3840 human genes, measured across 44 different tissues [1] (Geo [2] GDS829-GDS834). ‘AS co-expression’ of a gene was measured as the average pairwise Pearson correlation coefficient between the expression vectors of all its exon-junctions (see **Figure S10B**).

The expression of all exon-junctions of a particular gene was summarized (averaged) to form one vector representing the overall expression pattern of a gene. We measured ’GD co-expression’ of a GD family, i.e. the amount of co-expression amongst gene duplicates, as the average pairwise Pearson correlation coefficient between the gene expression vectors of all GD family members (see **Figure S10B**). For gene families of >80% sequence identity, the 3840 genes in the data set [1] did not provide enough families with more than two members to be suitable for further analysis. Thus we examined two other dataset with absolute expression values of human genes across 79 different tissues published by Su et al.[3](Geo [2] GDS596), and across 36 tissues published by Ge et al. [4] (Geo GDS1096).

As an additional measure we also used the number of different (binarized) expression patterns that are observed within a GD family or a group of splice variants. This measure can be normalized for the size of the GD family or the number of AS variants.

This table is divided into tables **A1-A4, B1-B2** and **C1-C2.** Tables **A1-A4** were obtained using Johnson et al.’s data [1] (Geo GDS829-834) of the expression of exon-junctions of 3,840 human genes across 44 tissues. Tables **B** and **C** were obtained using expression data sets (GeoGDS596 and GDS1096, respectively), with expression data of 12,270 genes across 79 and 36 tissues, respectively. Tables **A1** and **A2** show AS co-expression results (see **Figure S10B**, upper part); Tables **A3, A4, B,** and **C** show GD co-expression results (see **Figure S10B**, lower part).

##### A1. AS co-expression in all genes and the subset of known AS+ and AS- genes

The table shows AS co-expression for sets of genes from the data by Johnson et al. [1]. We examine the average pairwise Pearson correlation coefficient, the total number of expression vectors, the number of unique expression vectors and the ratio unique/total number of expression vectors for all genes and for genes of which AS+ is known from the AltSplice database [5]. Known AS+ genes have slightly lower Pearson correlation, and more unique expression vectors than AS- genes, consistent with their expression being more variable (less similar to each other). Thus, we can use the average pairwise Pearson correlation coefficient and the number of different row vectors as measures of co-expression between AS-variants.

|  | **Total** | **Average** | **Standard deviation** | **Minimum** | **First quartile** | **Median** | **Third quartile** | **Maximum** |
| --- | --- | --- | --- | --- | --- | --- | --- | --- |
| **All genes** |  |  |  |  |  |  |  |  |
| Average Pearson correlation between expression vectors | 2487 | **0.27** | 0.23 | -0.28 | 0.07 | **0.23** | 0.41 | 1.00 |
| Total number of expression vectors | 2693 | **10.93** | 17.18 | 1.00 | 4.00 | **7.00** | 13.00 | 577.00 |
| Unique number of expression vectors | 2487 | **7.51** | 8.89 | 1.00 | 3.00 | **5.00** | 9.00 | 262.00 |
| Ratio of unique/total number of expression vectors | 2487 | **0.73** | 0.24 | 0.02 | 0.55 | **0.75** | 1.00 | 1.00 |
| **AS+ (from AltSplice)** |  |  |  |  |  |  |  |  |
| Average Pearson correlation between expression vectors | 1171 | **0.25** | 0.21 | -0.18 | 0.08 | **0.21** | 0.38 | 1.00 |
| Total number of expression vectors | 1223 | **11.10** | 11.78 | 1.00 | 5.00 | **8.00** | 13.00 | 117.00 |
| Unique number of expression vectors | 1171 | **7.52** | 6.55 | 1.00 | 3.00 | **6.00** | 10.00 | 53.00 |
| Ratio of unique/total number of expression vectors | 1171 | **0.73** | 0.24 | 0.04 | 0.57 | **0.75** | 1.00 | 1.00 |
| **AS- (from AltSplice)** |  |  |  |  |  |  |  |  |
| Average Pearson correlation between expression vectors | 605 | **0.32** | 0.26 | -0.28 | 0.10 | **0.29** | 0.50 | 1.00 |
| Total number of expression vectors | 713 | **9.48** | 23.77 | 1.00 | 2.00 | **5.00** | 11.00 | 577.00 |
| Unique number of expression vectors | 605 | **7.07** | 12.12 | 1.00 | 3.00 | **5.00** | 8.00 | 262.00 |
| Ratio of unique/total number of expression vectors | 605 | **0.75** | 0.24 | 0.11 | 0.56 | **0.78** | 1.00 | 1.00 |

##### A2. AS co-expression in GD+ and GD- genes

The table shows AS co-expression for sets of genes from the data by Johnson et al. [1]. We compare the AS co-expression in genes with duplicates (GD+) to those genes without duplicates (GD-), defined by different sequence identity cutoffs.

|  | **Total** | **Average** | **Standard deviation** | **Minimum** | **First quartile** | **Median** | **Third quartile** | **Maximum** |
| --- | --- | --- | --- | --- | --- | --- | --- | --- |
|  |  |  |  |  |  |  |  |  |
| **40% GD+** |  |  |  |  |  |  |  |  |
| Average Pearson correlation between expression vectors | 707 | **0.29** | 0.24 | -0.17 | 0.09 | **0.26** | 0.45 | 1.00 |
| Total number of expression vectors | 770 | **9.58** | 11.51 | 1.00 | 3.00 | **7.00** | 11.00 | 130.00 |
| Unique number of expression vectors | 707 | **6.86** | 6.53 | 1.00 | 3.00 | **5.00** | 9.00 | 71.00 |
| Ratio of unique/total number of expression vectors | 707 | **0.74** | 0.24 | 0.08 | 0.56 | **0.76** | 1.00 | 1.00 |
| **40% GD-** |  |  |  |  |  |  |  |  |
| Average Pearson correlation between expression vectors | 1069 | **0.26** | 0.23 | -0.28 | 0.08 | **0.22** | 0.40 | 1.00 |
| Total number of expression vectors | 1166 | **11.11** | 20.09 | 1.00 | 4.00 | **7.00** | 13.00 | 577.00 |
| Unique number of expression vectors | 1069 | **7.70** | 10.08 | 1.00 | 3.00 | **6.00** | 10.00 | 262.00 |
| Ratio of unique/total number of expression vectors | 1069 | **0.73** | 0.24 | 0.04 | 0.56 | **0.75** | 1.00 | 1.00 |
|  |  |  |  |  |  |  |  |  |
| **60% GD+** |  |  |  |  |  |  |  |  |
| Average Pearson correlation between expression vectors | 328 | **0.26** | 0.24 | -0.12 | 0.04 | **0.23** | 0.42 | 1.00 |
| Total number of expression vectors | 356 | **8.44** | 10.61 | 1.00 | 3.00 | **6.00** | 10.00 | 130.00 |
| Unique number of expression vectors | 328 | **6.05** | 6.25 | 1.00 | 2.00 | **4.00** | 8.00 | 71.00 |
| Ratio of unique/total number of expression vectors | 328 | **0.73** | 0.26 | 0.08 | 0.53 | **0.77** | 1.00 | 1.00 |
| **60% GD-** |  |  |  |  |  |  |  |  |
| Average Pearson correlation between expression vectors | 1441 | **0.28** | 0.23 | -0.28 | 0.09 | **0.24** | 0.42 | 1.00 |
| Total number of expression vectors | 1573 | **10.99** | 18.38 | 1.00 | 4.00 | **8.00** | 13.00 | 577.00 |
| Unique number of expression vectors | 1441 | **7.68** | 9.33 | 1.00 | 3.00 | **6.00** | 10.00 | 262.00 |
| Ratio of unique/total number of expression vectors | 1441 | **0.73** | 0.24 | 0.04 | 0.57 | **0.75** | 1.00 | 1.00 |
|  |  |  |  |  |  |  |  |  |
| **80% GD+** |  |  |  |  |  |  |  |  |
| Average Pearson correlation between expression vectors | 138 | **0.19** | 0.21 | -0.12 | 0.00 | **0.12** | 0.31 | 0.87 |
| Total number of expression vectors | 152 | **6.63** | 6.03 | 1.00 | 3.00 | **5.00** | 8.00 | 39.00 |
| Unique number of expression vectors | 138 | **4.73** | 3.78 | 1.00 | 2.00 | **4.00** | 6.00 | 18.00 |
| Ratio of unique/total number of expression vectors | 138 | **0.70** | 0.28 | 0.17 | 0.50 | **0.74** | 1.00 | 1.00 |
| **80% GD-** |  |  |  |  |  |  |  |  |
| Average Pearson correlation between expression vectors | 1629 | **0.28** | 0.23 | -0.28 | 0.09 | **0.24** | 0.42 | 1.00 |
| Total number of expression vectors | 1775 | **10.85** | 17.84 | 1.00 | 4.00 | **7.00** | 13.00 | 577.00 |
| Unique number of expression vectors | 1629 | **7.60** | 9.13 | 1.00 | 3.00 | **6.00** | 10.00 | 262.00 |
| Ratio of unique/total number of expression vectors | 1629 | **0.74** | 0.24 | 0.04 | 0.57 | **0.76** | 1.00 | 1.00 |

##### A3. GD co-expression in GD+ genes

We examine the GD co-expression in the dataset from Johnson et al. [1]. The assessment of GD co-expression in this dataset is difficult, as not enough gene duplicates per family are present for useful comparisons. Most GD-families have only two or fewer members represented in the data (i.e. two or fewer expression vectors). For better assessment of GD co-expression, we also examined two other datasets; see tables **B.** and **C.**

|  | **Total** | **Average** | **Standard deviation** | **Minimum** | **First quartile** | **Median** | **Third quartile** | **Maximum** |
| --- | --- | --- | --- | --- | --- | --- | --- | --- |
| **40% GD+** |  |  |  |  |  |  |  |  |
| Average Pearson correlation between expression vectors | 105 | **0.24** | 0.33 | -0.37 | 0.00 | **0.18** | 0.42 | 1.00 |
| Total number of expression vectors | 105 | **2.20** | 0.43 | 2.00 | 2.00 | **2.00** | 2.00 | 4.00 |
| Unique number of expression vectors | 105 | **2.05** | 0.56 | 1.00 | 2.00 | **2.00** | 2.00 | 4.00 |
| Ratio of unique/total number of expression vectors | 105 | **0.93** | 0.17 | 0.50 | 1.00 | **1.00** | 1.00 | 1.00 |
| **60% GD+** |  |  |  |  |  |  |  |  |
| Average Pearson correlation between expression vectors | 43 | **0.39** | 0.42 | -0.32 | 0.00 | **0.29** | 0.81 | 1.00 |
| Total number of expression vectors | 43 | **2.09** | 0.29 | 2.00 | 2.00 | **2.00** | 2.00 | 3.00 |
| Unique number of expression vectors | 43 | **1.74** | 0.58 | 1.00 | 1.00 | **2.00** | 2.00 | 3.00 |
| Ratio of unique/total number of expression vectors | 43 | **0.83** | 0.24 | 0.50 | 0.50 | **1.00** | 1.00 | 1.00 |
| **80% GD+** |  |  |  |  |  |  |  |  |
| Average Pearson correlation between expression vectors | 14 | **0.47** | 0.46 | 0.00 | 0.00 | **0.41** | 0.95 | 1.00 |
| Total number of expression vectors | 14 | **2** | 0 | 2.00 | 2.00 | **2.00** | 2.00 | 2.00 |
| Unique number of expression vectors | 14 | **1.35** | 0.49 | 1.00 | 1.00 | **1.00** | 2.00 | 2.00 |
| Ratio of unique/total number of expression vectors | 14 | **0.67** | 0.24 | 0.50 | 0.50 | **0.50** | 1.00 | 1.00 |

##### A4. GD co-expression in AS+ and AS- genes

We examine GD co-expression in the dataset by Johnson et al. [1]. We define AS co-expression in several different ways: by i) a large number of unique expression vectors per gene (from the analysis in **A1** and **A2**); ii) small pairwise Pearson correlation coefficients amongst expression vectors of exon-junctions for a gene (from the analysis in **A1** and **A2**); and iii) the dataset published in AltSplice [5].

As mentioned in Table **A3**, the average total number of expression vectors (i.e. genes per family with expression data) and the unique number of expression vectors are very small for all sets, and make their comparison impossible. GD families were defined by 40% sequence identity.

|  | **Total** | **Average** | **Standard deviation** | **Minimum** | **First quartile** | **Median** | **Third quartile** | **Maximum** |
| --- | --- | --- | --- | --- | --- | --- | --- | --- |
| **i)** |  |  |  |  |  |  |  |  |
| **>6 unique AS expression rows** |  |  |  |  |  |  |  |  |
| Average Pearson correlation between expression vectors | 33 | **0.26** | 0.28 | -0.23 | 0.04 | **0.27** | 0.48 | 0.78 |
| Total number of expression vectors | 33 | **2.27** | 0.52 | 2.00 | 2.00 | **2.00** | 2.00 | 4.00 |
| Unique number of expression vectors | 33 | **2.24** | 0.50 | 2.00 | 2.00 | **2.00** | 2.00 | 4.00 |
| Ratio of unique/total number of expression vectors | 33 | **0.99** | 0.06 | 0.67 | 1.00 | **1.00** | 1.00 | 1.00 |
| **<=6 unique AS expression rows** |  |  |  |  |  |  |  |  |
| Average Pearson correlation between expression vectors | 62 | **0.21** | 0.33 | -0.37 | 0.00 | **0.09** | 0.33 | 1.00 |
| Total number of expression vectors | 62 | **2.19** | 0.40 | 2.00 | 2.00 | **2.00** | 2.00 | 3.00 |
| Unique number of expression vectors | 62 | **2.00** | 0.57 | 1.00 | 2.00 | **2.00** | 2.00 | 3.00 |
| Ratio of unique/total number of expression vectors | 62 | **0.91** | 0.19 | 0.50 | 1.00 | **1.00** | 1.00 | 1.00 |
| **ii)** |  |  |  |  |  |  |  |  |
| **AS co-expression with >0.5 average Pearson** |  |  |  |  |  |  |  |  |
| Average Pearson correlation between expression vectors | 26 | **0.34** | 0.34 | -0.32 | 0.06 | **0.19** | 0.54 | 1.00 |
| Total number of expression vectors | 26 | **2.27** | 0.45 | 2.00 | 2.00 | **2.00** | 2.00 | 3.00 |
| Unique number of expression vectors | 26 | **2.19** | 0.49 | 1.00 | 2.00 | **2.00** | 2.00 | 3.00 |
| Ratio of unique/total number of expression vectors | 26 | **0.97** | 0.12 | 0.50 | 1.00 | **1.00** | 1.00 | 1.00 |
| **AS co-expression with <= 0.5 average Pearson** |  |  |  |  |  |  |  |  |
| Average Pearson correlation between expression vectors | 69 | **0.19** | 0.30 | -0.37 | 0.00 | **0.14** | 0.31 | 1.00 |
| Total number of expression vectors | 69 | **2.20** | 0.44 | 2.00 | 2.00 | **2.00** | 2.00 | 4.00 |
| Unique number of expression vectors | 69 | **2.04** | 0.58 | 1.00 | 2.00 | **2.00** | 2.00 | 4.00 |
| Ratio of unique/total number of expression vectors | 69 | **0.93** | 0.18 | 0.50 | 1.00 | **1.00** | 1.00 | 1.00 |
| **iii)** |  |  |  |  |  |  |  |  |
| **AS+ families from AltSplice** |  |  |  |  |  |  |  |  |
| Average Pearson correlation between expression vectors | 58 | **0.23** | 0.33 | -0.26 | 0.00 | **0.14** | 0.35 | 1.00 |
| Total number of expression vectors | 1105 | **1.07** | 0.30 | 1.00 | 1.00 | **1.00** | 1.00 | 4.00 |
| Unique number of expression vectors | 58 | **2.16** | 0.56 | 1.00 | 2.00 | **2.00** | 2.00 | 4.00 |
| Ratio of unique/total number of expression vectors | 58 | **0.96** | 0.13 | 0.50 | 1.00 | **1.00** | 1.00 | 1.00 |
| **AS- families from AltSplice** |  |  |  |  |  |  |  |  |
| Average Pearson correlation between expression vectors | 47 | **0.28** | 0.37 | -0.37 | 0.00 | **0.21** | 0.48 | 1.00 |
| Total number of expression vectors | 634 | **1.09** | 0.32 | 1.00 | 1.00 | **1.00** | 1.00 | 3.00 |
| Unique number of expression vectors | 47 | **1.87** | 0.58 | 1.00 | 2.00 | **2.00** | 2.00 | 3.00 |
| Ratio of unique/total number of expression vectors | 47 | **0.87** | 0.22 | 0.50 | 0.67 | **1.00** | 1.00 | 1.00 |

##### B1. GD co-expression in GD+ families with and without AS analyzed in GDS596

The Geo-file GDS596.soft was analyzed as described above, using the AltSplice data as AS-reference, 600 units of signal intensity as cutoff for binarization and >80% sequence identity as cutoff for definition of GD+ families.

| **AS-/GD+** |  |  |  |  |  |  |  |
| --- | --- | --- | --- | --- | --- | --- | --- |
| Total entries |  |  | 526 |  |  |  |  |
|  | **Average** | **Standard dev**. | **Minimum** | **First Quartile** | **Median** | **Second Quartile** | **Maximum** |
| Total number of rows | **3.42** | 3.04 | 2.00 | 2.00 | **2.00** | 3.00 | 13.00 |
| Number of unique (binary) rows | **3.32** | 3.00 | 1.00 | 2.00 | **2.00** | 3.00 | 10.00 |
| Ratio Unique/Total number of rows | **0.97** | 0.11 | 0.33 | 1.00 | **1.00** | 1.00 | 1.00 |
| Average Pearson correlation | **0.40** | 0.27 | -0.35 | 0.23 | **0.38** | 0.58 | 1.00 |
| **AS+/GD+** |  |  |  |  |  |  |  |
| Total entries |  |  | 253 |  |  |  |  |
|  | **Average** | **Standard dev**. | **Minimum** | **First Quartile** | **Median** | **Second Quartile** | **Maximum** |
| Total number of rows | **2.45** | 1.24 | 2.00 | 2.00 | **2.00** | 2.00 | 11.00 |
| Number of unique (binary) rows | **2.38** | 1.20 | 1.00 | 2.00 | **2.00** | 2.00 | 8.00 |
| Ratio Unique/Total number of rows | **0.97** | 0.10 | 0.33 | 1.00 | **1.00** | 1.00 | 1.00 |
| Average Pearson correlation | **0.34** | 0.27 | -0.35 | 0.18 | **0.34** | 0.50 | 1.00 |

##### B2. Correlation between fraction of AS+ per GD family and measures of GD co-expression.

| **Total data points 779** | **Pearson’s R** |
| --- | --- |
| **Fraction of genes with AS+ in GD family correlated with** |  |
| Number of unique expression rows | -0.21 |
| Ratio Unique/Total number of rows | 0.20 |
| Average Pearson correlation between expression vectors | -0.13 |

##### C1. GD co-expression in GD+ families with and without AS analyzed in GDS1096

The Geo-file GDS1096.soft was analyzed as described above, using the AltSplice [5] data as AS-reference, 600 units of signal intensity as cutoff for binarization and >80% sequence identity as cutoff for definition of GD+ families. All trends are the same irrespective of the parameters. The results are qualitatively the same as in table **B**.

| **AS-/GD+** |  |  |  |  |  |  |  |
| --- | --- | --- | --- | --- | --- | --- | --- |
| Total entries |  |  | 526 |  |  |  |  |
|  | **Average** | **Standard dev.** | **Minimum** | **First Quartile** | **Median** | **Second Quartile** | **Maximum** |
| Total number of rows | **3.38** | 2.55 | 2.00 | 2.00 | **2.00** | 3.00 | 13.00 |
| Number of unique (binary) rows | **1.87** | 1.16 | 1.00 | 1.00 | **2.00** | 2.00 | 6.00 |
| Ratio Unique/Total number of rows | **0.64** | 0.29 | 0.15 | 0.50 | **0.50** | 1.00 | 1.00 |
| Average Pearson correlation | **0.25** | 0.34 | -0.68 | 0.07 | **0.27** | 0.56 | 1.00 |
| **AS+/GD+** |  |  |  |  |  |  |  |
| Total entries |  |  | 253 |  |  |  |  |
|  | **Average** | **Standard dev.** | **Minimum** | **First Quartile** | **Median** | **Second Quartile** | **Maximum** |
| Total number of rows | **2.61** | 1.25 | 2.00 | 2.00 | **2.00** | 2.00 | 11.00 |
| Number of unique (binary) rows | **1.76** | 0.98 | 1.00 | 1.00 | **2.00** | 2.00 | 6.00 |
| Ratio Unique/Total number of rows | **0.69** | 0.26 | 0.27 | 0.50 | **1.00** | 1.00 | 1.00 |
| Average Pearson correlation | **0.16** | 0.32 | -0.68 | -0.07 | **0.14** | 0.36 | 1.00 |

##### C2. Pearson correlation between fraction of AS+ per GD family and measures of GD co-expression

| **Total data points 779** | **Pearson’s R** |
| --- | --- |
| Fraction of genes with AS+ in GD family correlated with |  |
| Number of unique expression rows | 0.04 |
| Ratio Unique/Total number of rows | 0.22 |
| Average Pearson correlation between expression vectors | -0.23 |

**REFERENCES**

1. Johnson JM, Castle J, Garrett-Engele P, Kan Z, Loerch PM, et al. (2003) Genome-wide survey of human alternative pre-mRNA splicing with exon junction microarrays. Science 302: 2141-2144.

2. Barrett T, Suzek TO, Troup DB, Wilhite SE, Ngau WC, et al. (2005) NCBI GEO: mining millions of expression profiles--database and tools. Nucleic Acids Res 33: D562-566.

3. Su AI, Wiltshire T, Batalov S, Lapp H, Ching KA, et al. (2004) A gene atlas of the mouse and human protein-encoding transcriptomes. Proc Natl Acad Sci U S A 101: 6062-6067.

4. Ge X, Yamamoto S, Tsutsumi S, Midorikawa Y, Ihara S, et al. (2005) Interpreting expression profiles of cancers by genome-wide survey of breadth of expression in normal tissues. Genomics 86: 127-141.

5. Thanaraj TA, Stamm S, Clark F, Riethoven JJ, Le Texier V, et al. (2004) ASD: the Alternative Splicing Database. Nucleic Acids Res 32: D64-69.
