# Supplementary material for: The (In)dependence of Alternative Splicing and Gene Duplication
Source: PLoS Comput Biol. 2007 Mar 2;3(3):e33. doi: 10.1371/journal.pcbi.0030033 (PMC1808492; doi:10.1371/journal.pcbi.0030033)
Supplement: Supplementary file 5 [file pcbi.0030033.st005.doc]

## Table S5. Overview of the data set employed in the protein

## sequence/structure analysis.

The table shows the number of genes with AS, and the number of multiple gene families, together with the respective number of sequences. Information on AS was taken from SwissProt [1]. The data are provided for human and mouse, for 40 % and 80 % sequence identity clusters.

|  | **Number of genes with AS in multigene families** | **Total number of isoforms** | **Number of multigene families** | **Total number of proteins in gene families** |
| --- | --- | --- | --- | --- |
| **Human 40%** | 798 | 2248 | 558 | 1163 |
| **Mouse 40%** | 282 | 768 | 222 | 623 |
| **Human 80%** | 134 | 362 | 84 | 214 |
| **Mouse 80%** | 31 | 91 | 22 | 57 |

**REFERENCES**

1. Bairoch A, Apweiler R (2000) The SWISS-PROT protein sequence database and its supplement TrEMBL in 2000. Nucleic Acids Res 28: 45-48.
